# Favorable changes in health- and work-related factors and subsequent work ability in midlife and older social and health care employees

**DOI:** 10.1007/s00420-025-02200-4

**Published:** 2026-02-11

**Authors:** Joonas Poutanen, Mikko Härmä, Tea Lallukka, Matti Joensuu, Eija Haukka, Aki Koskinen, Jenni Ervasti, Rahman Shiri

**Affiliations:** 1 Finnish Institute of Occupational Health, P.O. Box 18, 00032 Helsinki, Finland; 2 Department of Public Health, University of Helsinki, Helsinki, Finland

**Keywords:** Alcohol, Demands, Exercise, Psychological distress, Sleep, Smoking

## Abstract

**Objective:**

This quasi-experimental study aimed to examine whether favorable changes in health- and work-related factors improve work ability of midlife and older workers.

**Methods:**

The study included 2,312 Finnish social and health care employees (89.4% women, mean age 51.5 ± 5.8 years) who participated in repeated surveys between 2017 and 2022. Propensity score weighting and generalized linear models were applied to estimate the associations of a one standard deviation favorable change in health and lifestyle factors, psychosocial work environment and working hour characteristics with perceived work ability.

**Results:**

Favorable changes in health-related factors including improvement in sleep quality (RR 2.03, 95% CI 1.47–2.82), reduction in psychological distress (RR 1.68, 95% CI 1.24–2.30), increase in leisure-time physical activity (1.53, 95% CI 1.13–2.08), quitting smoking (RR 1.58, 95% CI 1.01–2.48), decrease in alcohol intake (RR 2.21, 95% CI 1.54–3.17) were associated with improved work ability. Of work-related factors, reduction in job demands (RR 1.51, 95% CI 1.12–2.03) and job demand-control ratio (RR 1.55, 95% CI 1.11–2.16), and increase in job control (RR 1.49, 95% CI 1.05–2.13) and job rewards (RR 1.60, 95% CI 1.20–2.13) were associated with improved work ability.

**Conclusions:**

Positive changes in modifiable health- and work-related risk factors can improve work ability of midlife and older social and health care employees. These identified risk factors are essential in design and implementation of multidimensional interventions aimed at supporting extended workforce participation among older workers.

## Introduction

As populations in developed countries continue to age, a growing segment of the workforce is comprised of midlife and older workers (OECD 2024b). The combination of an aging population and declining fertility rates presents societal challenges, including labor shortages. On an individual level, aging causes inevitable changes in mental and physical functioning, yet it does not necessarily result in reduced work performance or productivity (Silverstein 2008). To support extended workforce participation, maintaining adequate work ability is crucial. Several work ability theories exists (De Rijk 2013; Ilmarinen and Tuomi 1992; Tengland 2011) with the most established framework (Ilmarinen and Tuomi 1992) consisting of individual resources (health, functional capacities, competence, values, attitudes and motivation), factors related to work (management, environment and community), and the external environment (such as family, social networks and society). Poor work ability is linked to increased risks of sickness absence (Palmlof et al. 2019), disability retirement (Alavinia et al. 2009; Jaaskelainen et al. 2016), early old-age retirement (Salonen et al. 2003) and even mortality (Elovainio et al. 2022).

Observational studies have identified risk factors for decreased work ability. A systematic review (van den Berg et al. 2009) of 14 cross-sectional studies and six longitudinal studies found that older age and health-related factors, including insufficient leisure-time physical activity and obesity, are risk factors for poor work ability. Additionally, unhealthy diet and smoking were found to be risk factors in a longitudinal study (De Bortoli et al. 2021). Work-related factors such as lack of autonomy and high physical and mental work demands are also known to be associated with poor work ability (van den Berg et al. 2009). Frequent or long-term sickness absence is generally considered as a sign of reduced work ability. Studies suggest that risk factors for sickness absence may vary between younger and older workers. For instance, a prospective cohort study (Andersen et al. 2021) observed that older workers facing high physical work demands experienced higher rates of long-term sickness absence compared to their younger counterparts. Moreover, a quasi-experiment (Shiri et al. 2024) showed that reducing job demands and increasing job control lowered the risk of sickness absence among public sector employees aged 50 and older, while increasing job rewards benefited younger employees. These findings imply that risk factors for decreased work ability may differ between younger and older workers. Therefore, the relationship between different types of risk factors and work ability needs further exploration specifically among older workers who are at increased risk of work disability (Leijten et al. 2015). Promoting work ability effectively, especially in the later stages of a working career, is essential to mitigate risks and support prolonged workforce participation. Despite its importance, only few randomized controlled trials (RCTs) have assessed interventions aimed at improving work ability among midlife and older workers. For example, physical exercise interventions have shown modest effectiveness (Andersen et al. 2015; Stenner et al. 2020). However, given the complex nature of work ability (De Rijk 2013; Ilmarinen and Tuomi 2004; Tengland 2011), the benefits of such single component health promotion interventions are limited. Multicomponent interventions addressing individual resources beyond health, such as skill development, along with organizational factors like job demands and working time arrangements, are crucial for significantly improving work ability. However, to date, only a limited number of intervention studies have included both work-related components and older worker populations (Morelock et al. 2017; Shiri et al. 2021b; Vuori et al. 2019), and their effects on work ability have been mixed. This research gap may be due to the complexity of implementing organizational-level interventions compared to single-component efficacy experiments.

Quasi-experimental design offers a way to address challenges associated with observational studies (e.g., confounding factors) and intervention studies (e.g. implementation of complex interventions). In quasi-experiments, observational data with repeated measurements of the same participants are analyzed, mimicking RCTs (Lallukka and Shiri 2020; Ross et al. 2015). These studies are often more feasible to conduct than RCTs and confounding can be limited (Lallukka and Shiri 2020). Confounding factors can be reduced with propensity score weighting, which mimics the randomization process of the RCTs by balancing the distribution of covariates between exposed and unexposed groups (Ross et al. 2015). Quasi-experimental design further enables to examine whether a change in exposure results in a change in the outcome of interest (Lallukka and Shiri 2020).

In addition to individual characteristics and work-related risk factors that contribute to decreased work ability, employment in the public sector potentially further elevates the risk. Public sector employees have been found to experience higher rates of both full- and part-time sickness absence compared to their counterparts in the private sector (Hartikainen et al. 2022). This highlights the importance of effective work ability promotion especially among public sector employees. The aim of this quasi-experiment study was to examine whether favorable changes in health- and work-related factors can improve perceived work ability among social and health care employees working in the public sector aged 40 years and older. Health-related factors included overweight or obesity, sleep quality and psychological distress, physical activity, smoking status, and alcohol consumption, and work-related factors included job demands, job control, effort at work, job rewards, and worktime control, weekly working hours, proportion of shift intervals of less than 11 h during the year, and proportion of single free days to all free days during the year.

## Methods

### Population

**Fig. 1:**
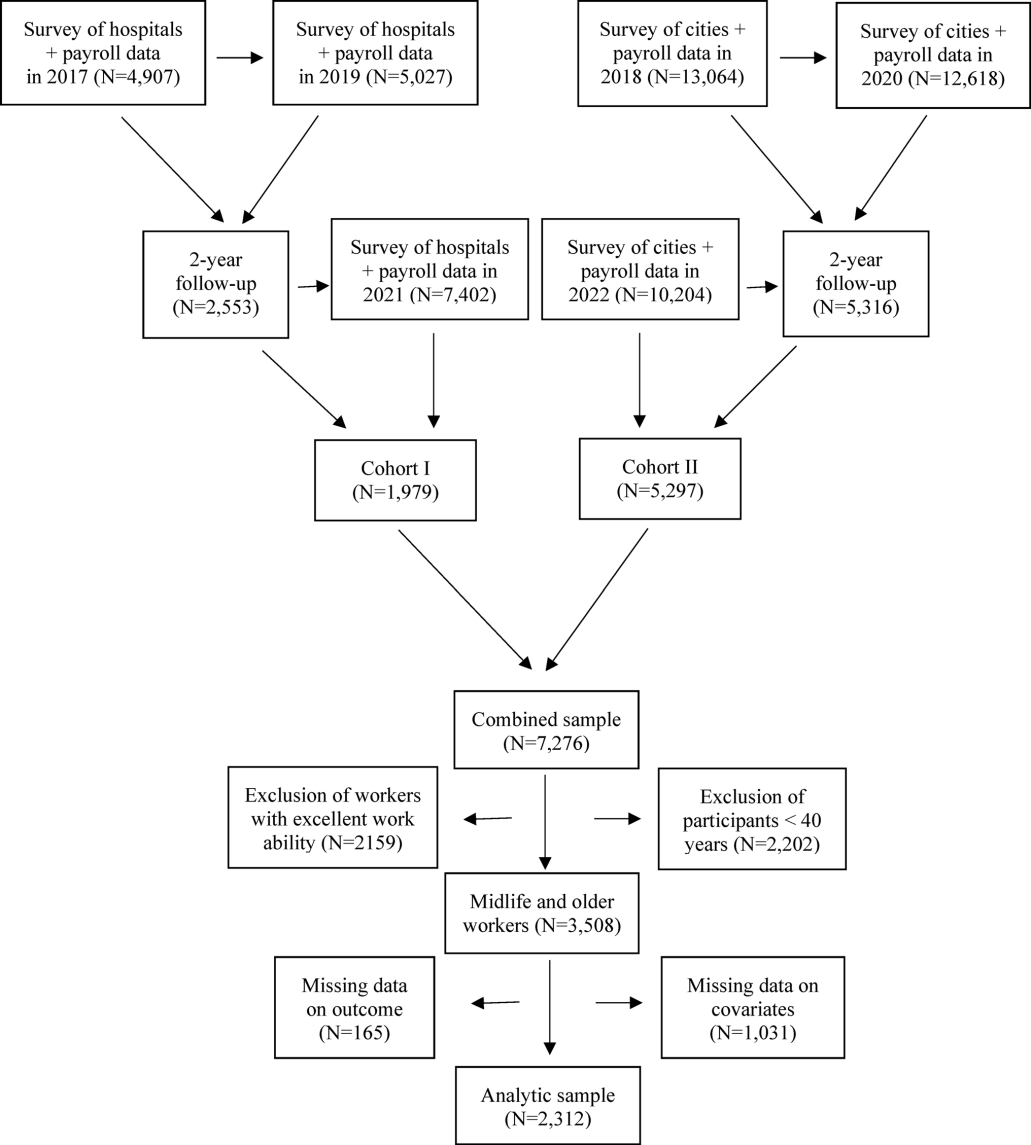
Flow chart of the study population, the finnish public sector (FPS) study

The current study utilized the Working Hours in the Finnish Public Sector (WHFPS) payroll database and linked the data with the Finnish Public Sector (FPS) surveys, an ongoing prospective dynamic cohort of employees from the 10 hospital districts and the 11 cities (Ervasti et al. 2023). The current study sample consisted of 7,276 social and health care employees from three hospital districts and five cities who answered the survey at baseline in 2017 or 2018 and at 2 years follow up in 2019 or 2020 (Fig. 1). From those, workers with excellent (score 9 and 10) work ability at baseline (N = 2,159) were excluded. The final analytical sample (N = 2312) consisted of workers aged 40 years or older who had complete data on background characteristics and baseline work ability, as well as follow-up data on work ability from 2021 or 2022. The ethics committee of the Hospital District of Helsinki and Uusimaa has approved the study (HUS/1210/2016).

### Health-related factors

Health-related factors were measured with overweight or obesity, sleep quality and psychological distress. Overweight and obesity were evaluated with body mass index (BMI), which was calculated from self-reported weight and height. BMI between 25.0 and 29.9 kg/m^2^ was defined as overweight and BMI ≥ 30.0 kg/m^2^ as living with obesity. Sleep problems were assessed with a four-item Jenkins Sleep Scale (Juhola et al. 2021). Psychological distress was measured with the 12-item General Health Questionnaire (GHQ-12) (Goldberg et al. 1997). Health-related lifestyle factors included physical activity, smoking status, and alcohol consumption. Intensity of leisure-time physical activity in the past 12 months were measured using questions on walking, brisk walking, jogging and running. Metabolic Equivalent (MET) index was computed by multiplying the MET value of each activity intensity by the time spent on that activity and adding up the METs of the four activities. Physical activity intensity was defined as moderate (≥ 14 MET-hours of moderate-intensity activities with no vigorous activities) and vigorous (≥ 14 MET-hours, including some vigorous activity such as jogging or running) activity (Shiri et al. 2020b). Smoking status was classified into never smokers, former smokers and current smokers. Alcohol consumption was assessed by asking about the weekly frequency of beer and wine intake and the monthly frequency of spirit intake. The information was converted into grams of pure alcohol consumed per week following previous procedures: one unit of pure alcohol (12 g) is equal to a 33-cl bottle of beer, 12-cl glass of wine, or a 4-cl of spirit (Kouvonen et al. 2007).

### Work-related factors

Work-related factors consisted of psychosocial factors at work including job demands, job control, effort at work, job rewards, and worktime control. Job demands were measured with five items and job control with nine items (Ervasti et al. 2023). Job demand-control ratio was calculated using the demands score divided by the control score (Piantella et al. 2021), then multiplying by 0.556. This correction factor was derived by dividing the number of job demands items by number of job control items (Lau 2008). Effort at work was measured with one item: ‘how much do you invest your talents and resources in the work?’ (Juvani et al. 2014). Job rewards was measured with three items that asked how much participants received compensation for their work in terms of (1) income, employment benefits and other monetary rewards, (2) recognition and appreciation and (3) personal satisfaction (Juvani et al. 2014). Effort-reward ratio was calculated using ratio of ‘(effort score/reward score) × 0.333’. The correction factor (of 0.333) was derived by dividing the effort item by number of job rewards items (Lau 2008). Worktime control was measured with seven items (Ala-Mursula et al. 2004; Ervasti et al. 2023).

Also, work-related factors included the following working hour characteristics: register-based weekly working hours, proportion of shift intervals of less than 11 h during the year, and proportion of single free days to all free days during the year. Such irregular working-hour characteristics are known to be associated with various adverse health related outcomes (Gan et al. 2015; Wang et al. 2011) and sickness absence (Shiri et al. 2021a).

### Covariates

The questionnaire collected data on several variables in addition to exposures: age, gender, marital status, supervisory role, type of work contract (full-time or part-time), having a child younger than seven years, years at the current job, years in shift work, types of work shifts, sleep duration, self-rated general health, satisfaction with employee development, employee influence on planning work changes, average length of all shifts during the year (hours), average number of consecutive daily work shifts during the year, average number of consecutive evening shifts during the year, average time between work shifts (hours) during the year, average number of consecutive night shifts during the year, proportion of recovery periods of less than 28 h after the last night shift during the year, proportion of recovery periods of less than 48 h after the last night shift during the year, and the proportion of weekly recovery periods of less than 48 h.

### Work ability

Work Ability Score (WAS), a single item from the Work Ability Index (WAI) was used to measure work ability. In WAS, perceived current work ability is compared to the lifetime best on a 0 to 10 scale. Studies have shown that WAS has good predictive validity (Ahlstrom et al. 2010; Lundin et al. 2017) and reliability (van Dinter et al. 2025). Work ability was classified into four categories: poor (score 0 − 5), moderate (score 6–7), good (score 8–9), and excellent (score 10) (Kinnunen and Nätti 2018). To mimic a randomized controlled trial (RCT), workers with poor or moderate work ability at baseline (2017 or 2018) were included and followed them over time to assess improvements in work ability (Fig. 1). Since a third of the study sample had a baseline score of 8, the at-risk population was defined as those with scores ranging from 0 to 8. The outcome was defined as achieving good or excellent work ability, with scores of 9 to 10 at follow-up.

### Statistical analysis

We defined the intervention as at least one standard deviation (SD) change in exposure between 2017/2018 and 2019/2020. To estimate the probability of experiencing an increase or decrease in exposure (hereafter referred to as intervention) for each participant, a propensity score was calculated using multilevel mixed-effects logistic regression, with employees nested within units.

Variance Inflation Factor (VIF) was used to assess multicollinearity among covariates. Variables with VIF of 5 or higher were excluded from the propensity score estimation including: having a child aged 7–18 years, 3-category BMI variable (underweight/normal, overweight, obese), the average length of all night shifts, the proportion of weekly recovery periods < 35 h and the proportion of weekend work. Finally, the propensity score included the following baseline characteristics and outcome variables from 2017/2018: age (both continuous and categorical), gender, marital status, supervisory role, type of work contract (full-time or part-time), having a child younger than seven years, years at the current job, years in shift work, types of work shifts, smoking status, weekly alcohol consumption, leisure time physical activity (MET, continuous), body mass index (continuous), sleep duration, sleep quality, psychological distress, self-rated general health, work ability (both continuous and categorical), job demands, job control, effort at work, job rewards, worktime control, satisfaction with employee development, employee influence on planning work changes, weekly working hours, average length of all shifts during the year (hours), average number of consecutive daily work shifts during the year, average number of consecutive evening shifts during the year, average time between work shifts (hours) during the year, proportion of shift intervals of less than 11 h during the year, proportion of recovery periods of less than 28 h after the last night shift during the year, proportion of recovery periods of less than 48 h after the last night shift during the year, proportion of weekly recovery periods of less than 48 h, and proportion of single free days to all free days.

To estimate the inverse probability of treatment weight for each employee, we utilized the propensity score. To minimize variability and bias, we stabilized these weights (Shiri et al. 2020a; Xu et al. 2010). To compare improvement in work ability in 2021/2022 between the intervention and control groups, we employed a generalized linear model with a binomial distribution and a log link function, using the stabilized inverse probability of treatment weights as a weighting factor. The P values were adjusted using the Bonferroni correction to control for type I error due to multiple testing. The corrected significance level was set at 0.0031 (α = 0.05/16 = number of exposures). All analyses were conducted using Stata, version 18 (StataCorp, College Station, TX, USA).

## Results

In this dynamic cohort, 17,971 participants responded at baseline (2017/2018) (Fig. 1). Of these, 7,869 (43.8%) completed the 2 years follow-up (2019/2020), and 7,276 (40.5%) responded in 2021/2022. Participants with excellent work ability (N = 2,159), those younger than 40 years (N = 2,202) and individuals with missing data on covariates (N = 1,031) or outcome (N = 165) were excluded. The final study population comprised 2,312 participants, with a mean age of 51 years. Women represented 89% of the participants (Table 1). Most participants worked full-time (93.2%), and 45.9% reported having a regular day job. Overall, 53.4% rated their general health as good or fairly good. Of the participants, 24.1% were from three hospital districts, while 75.9% were from five cities.

**Table 1:** The baseline characteristics of the study population (N = 2,312) consisting of midlife and older social and health care employees

Characteristic	%	Mean ± SD
Age (years)		51.1 ± 5.8
40–49	37.6	
50–59	58.2	
60–64	4.2	
Female gender	89.4	
Marital status		
Single	14.0	
Married	53.7	
Cohabiting	15.0	
Divorced or separated or widowed	17.3	
Leisure time physical activity		
Moderate	49.8	
Strenuous	19.2	
Smoking		
Past	24.6	
Current	15.9	
Body mass index		
Overweight (25.0–29.9 kg/m^2^)	37.0	
Obesity (≥ 30.0 kg/m^2^)	28.7	
Work contract		
Full-time	93.2	
Part-time	6.8	
Being a supervisor	7.3	
Type of shift work		
Regular day work	45.9	
Shift work without night shifts	28.1	
Shift work with night work	22.9	
Regular night work	0.4	
Other irregular work	2.7	
Number of years in the current job		18.9 ± 11.4
Duration of shift work (years)		12.0 ± 11.3
Weekly working hours		34.4 ± 3.7
Percentage of short shift interval (< 11 h)		8.0 ± 11.0
Percentage of long night shifts (≥ 10 h)		3.4 ± 9.6
Current work ability		
Poor (score 0–5)	11.9	
Moderate (score 6–8)	88.1	
Self-rated general health		
Good	11.0	
Fairly good	42.4	
Average	38.6	
Fairly bad	7.7	
Bad	0.3	

During the follow-up period, 13.5% of participants (N = 311) achieved excellent work ability (score 9–10). Among the at-risk group, the proportion of participants showing favorable changes in health-related factors ranged from 3.4 to 25.3%. Improvement in sleep quality by at least one standard deviation (SD) was significantly associated with improved work ability [Risk Ratio (RR) 2.03, 95% Confidence Interval (CI) 1.47–2.82; Table 2]. Similarly, a reduction in psychological distress by at least one SD was associated with improved work ability (RR 1.68, 95% CI 1.24–2.30). However, a decrease in BMI among participants with overweight or obesity was not associated with improvement in work ability. Among health-related lifestyle factors, an increase in physical activity (measured in METs) by at least one SD was associated with improved work ability (RR 1.53, 95% CI 1.13–2.08). Additionally, smoking cessation (RR 1.58, 95% CI 1.01–2.48) and a reduction in alcohol consumption by one SD (RR 2.21, 95% CI 1.54–3.17) were associated with improved work ability.

**Table 2:** The associations of favorable changes in health-related factors and improved perceived work ability (WAS score 9–10) among midlife and older social and health care employees

Characteristic	Sample	% of outcome	RR	95% CI	P
At least 1 SD increase in MET among inactive (14.8 units) or moderately active (19.9 units) participants at baseline					
No	1,355	12.2	1		
Yes	230	18.6	1.53	1.13 − 2.08	0.007
Quitting smoking					
No	2,174	13.3	1		
Yes	77	21.0	1.58	1.01 − 2.48	0.046
At least 1 SD decrease (51 g) in alcohol intake among drinkers					
No	1,649	10.9	1		
Yes	110	24.0	2.21	1.54 − 3.17	< 0.001
At least 1 SD decrease (≥ 2.3 kg/m^2^) in BMI among overweight or obese participants at baseline					
No	1,360	13.5	1		
Yes	96	13.5	1.00	0.59 − 1.69	
At least 1 SD improvement in sleep quality (Jenkins Sleep Scale) among participants with value above the median level at baseline					
No	929	8.6	1		
Yes	287	17.6	2.03	1.47 − 2.82	< 0.001
At least 1 SD reduction in psychological distress among participants with value above the median level at baseline					
No	875	10.7	1		
Yes	296	18.1	1.68	1.24 − 2.30	0.001

Abbreviations: BMI = Body Mass Index, MET = Metabolic Equivalent of Task

Favorable changes in psychosocial factors at work were observed in 18.4% to 30.6% of the at-risk group. A reduction of at least one SD in job demands (RR 1.51, 95% CI 1.12–2.03) and in the job demand-control ratio (RR 1.55, 95% CI 1.11–2.16), as well as an increase of at least one SD in job control (RR 1.49, 95% CI 1.05–2.13) and job rewards (RR 1.60, 95% CI 1.20–2.13), were all associated with improved work ability (Table 3). No significant associations were found between changes in work effort, effort-reward ratio, or worktime control and work ability. Regarding working hour characteristics, favorable changes were observed in 8.1% to 33.5% of the at-risk group. However, changes in weekly working hours, the proportion of short shift intervals (< 11 h), and the proportion of single free days were not associated with improved work ability.

**Table 3:** The associations of favorable changes in work-related factors and improved perceived work ability (WAS score 9–10) among midlife and older social and health care employees

Characteristic	Sample	% of outcome	RR	95% CI	P
At least 1 SD reduction in job demands among participants with demands above the median level at baseline					
No	937	12.7	1		
Yes	276	19.1	1.51	1.12 − 2.03	0.007
At least 1 SD increase in job control among participants with control below the median level at baseline					
No	968	10.5	1		
Yes	224	15.7	1.49	1.05 − 2.13	0.026
At least 1 SD reduction in job demand-control ratio among participants with value above the median level at baseline					
No	850	11.6	1		
Yes	229	18.0	1.55	1.11 − 2.16	0.010
At least 1 SD reduction in work effort among participants with value above the median level at baseline					
No	1,499	13.9	1		
Yes	446	13.4	0.97	0.74 − 1.26	
At least 1 SD increase in job rewards among participants with rewards below the median level at baseline					
No	937	10.6	1		
Yes	413	16.9	1.60	1.20 − 2.13	0.001
At least 1 SD reduction in effort-reward ratio among participants with value above the median level at baseline					
No	846	12.2	1		
Yes	191	12.4	1.02	0.67 − 1.54	
At least 1 SD increase in worktime control among participants with value below the median level at baseline					
No	934	11.7	1		
Yes	214	13.2	1.13	0.77 − 1.66	
At least 1 SD increase in weekly working hours					
No	1,984	13.3	1		
Yes	174	10.6	0.79	0.51 − 1.24	
At least 1 SD reduction in percentage of short shift interval (< 11 h) among participants with value above 0% at baseline					
No	767	13.9	1		
Yes	386	10.3	0.74	0.52 − 1.04	
At least 1 SD increase in percentage of single free days					
No	1,777	13.2	1		
Yes	168	16.5	1.25	0.87 − 1.80	

## Discussion

This quasi-experiment study found that favorable changes in several modifiable health- and work-related factors can improve work ability among midlife and older social and health care employees with less than excellent work ability at baseline. In contrast, changes in working hour characteristics were not associated with improved work ability. Effective lifestyle changes include increasing physical activity, quitting smoking and reducing alcohol consumption. Additionally, improved sleep quality and reduced psychological distress were associated with improved work ability. Reduction in job demands, alongside increases in job control and job rewards also contributed to improved work ability. These findings underscore the importance of implementing multi-component interventions, including health promotion and improvement of sleep, mental health and psychosocial factors at work for midlife and older workers.

The beneficial effect of physical activity on work ability observed in this study aligns with previous research. RCTs have demonstrated that interventions incorporating physical exercise can modestly improve work ability among midlife and older workers (Andersen et al. 2015; Stenner et al. 2020). Similarly, our results corroborate earlier prospective cohort study reporting an association between smoking and poor work ability (De Bortoli et al. 2021). Both smoking (Canivet et al. 2013; Harkonmaki et al. 2007) and alcohol consumption (Morois et al. 2016; Salonsalmi et al. 2012) have also been identified as risk factors for disability retirement, a consequence of poor work ability. It can be hypothesized that underlying health problems may have triggered some participants to adopt healthier behavior, leading to enhanced health and, consequently, improved work ability.

Among other health-related factors, improved sleep quality and reduced psychological distress were both associated with improved work ability, consistent with previous evidence. Prospective cohort studies have shown that better sleep quality is linked to improved work ability among nurses (Camerino et al. 2008), while sleep problems are associated with an increased risk of disability retirement among midlife and older workers (Hale et al. 2017; Lallukka et al. 2014). Similarly, psychological distress (Leijon et al. 2017) have been identified as a predictor of poor work ability in a 7-year follow-up study of the general working population.

Surprisingly, favorable changes in BMI among individuals with overweight or obesity had no effect on work ability. This finding contrasts with previous longitudinal studies indicating that increased BMI is linked to decreased work ability (van de Ven et al. 2022; van den Berg et al. 2009). Notably, only a small proportion of participants with overweight or obesity experienced a weight reduction of at least one SD during the follow-up period. Furthermore, due to the limited sample size, we were unable to restrict our analysis to individuals with obesity at baseline, which may partly explain the lack of association. Obesity has been shown to pose a greater risk to work ability than overweight (Andersen et al. 2017). Another explanatory factor of lack of association could be the presence of metabolically healthy obesity. Although obesity is a risk factor for several diseases and contributes to a reduced life expectancy, significant proportion of those living with obesity could be metabolically healthy, which is more prevalent among women (Bluher 2020). In our study, nearly 90% of participants were women and only a small proportion reported impaired health at baseline, suggesting the possible presence of metabolically healthy obesity within the sample.

Regarding psychosocial factors at work, we found that reduction in job demands and increase in job control were associated with improved work ability. These results strengthen previous evidence about the importance of these factors on work ability. For example, a systematic review (van den Berg et al. 2009) showed that high physical and mental work demands were associated with poor work ability. Also, several studies have identified the association between low job control and disability retirement among midlife and older workers (Carlsson et al. 2024; Lahelma et al. 2012; Robroek et al. 2013). However, conflicting results compared to our findings exist regarding the benefits of increasing job rewards. A quasi-experiment study (Shiri et al. 2024) found that increasing job rewards benefitted only workers under 50 years of age in terms of risk of sickness absence, an outcome which can be considered as a consequence of decreased work ability, while our results indicate that increasing job rewards is beneficial for work ability also among midlife and older workers. Based on our results, psychosocial factors at work including job demands, job control and rewards should be especially considered in work ability promotion in the mid and later stages of working careers.

Changes in other psychosocial factors at work including reduction in work effort, reduction in effort-reward ratio, and increase in worktime control had no effect on work ability. To our knowledge, there is a lack of research regarding the associations between these factors and work ability, but studies with work (dis)ability related outcomes, such as sickness absence and disability retirement exist with findings differing from our results. For example, a longitudinal study (Juvani et al. 2014) found that effort-reward imbalance was associated with an increased risk of disability retirement. Also, age-adjusted results of a previous follow-up study among representative sample of Finnish employees suggested that high worktime control decreased the risk of long-term sickness absence (Natti et al. 2015). However, there is no clear association between worktime control and health or well-being according to a previous systematic review (Nijp et al. 2012). The inconsistent results may be explained by the different outcomes, samples and designs used in studies.

Furthermore, we found that favorable changes in register based working hour characteristics were not associated with improved work ability, although irregular working hours are known to be associated with adverse health conditions (Gan et al. 2015; Wang et al. 2011) and sickness absence (Shiri et al. 2021a). The result combined with the null result on worktime control indicates that working hour characteristics might have a smaller role in perceived work ability among midlife and older workers compared to health- and other work-related factors. However, the result should be treated with caution as the lack of associations could be partly explained by the relatively small sample which could decrease statistical power.

### Strengths and limitations

This study has several strengths. We analyzed a longitudinal study using a quasi-experimental design to mimic a RCT enabling us to balance the distribution of covariates between the intervention and control groups and thus reduce confounding. Information on working hours was derived from detailed pay-roll registry data. The study focused on participants with decreased work ability (WAS scores 0 − 8) who were considered in need of interventions aimed at improving their work ability. Workers with excellent work ability were excluded, as they were not the target population for such interventions. Participants were followed over the study period to assess improvements in work ability, with the attainment of excellent work ability (WAS score 9 or 10) serving as the primary outcome. This approach allowed us to construct prospective study design, including only participants who were free from the outcome of interest at baseline and tracking the incidence of new cases during follow-up. Additionally, we excluded moderately correlated covariates from the propensity score model. Nevertheless, a sensitivity analysis that included these covariates showed that their inclusion did not affect the results.

However, the study has limitations. Although we were able to assess several health- and work-related risk factors, we did not collect data on all factors related to work ability such as competence and motivation. Also, due to the observational study design, it is not possible to confirm causal relationships between the risk factors and work ability, or to assess methods that were used to increase or decrease exposures. The study design enabled us to evaluate the improvement in work ability, although maintaining good or excellent work ability is equally important, especially among older workers.

Missing data on outcome or covariates resulted in the exclusion of 34% of the potential analytic sample. Consequently, the sample size was insufficient to ensure adequate statistical power for evaluating certain interventions, particularly because there were only a small number of workers who exhibited favorable changes. Furthermore, after applying Bonferroni correction to adjust P values for multiple testing and control for type I error, the associations between quitting smoking, increased job control, or a reduced job demand-control ratio and improved work ability were no longer statistically significant. However, this does not necessarily indicate the absence of true associations. The limited sample size likely contributed to the inability to detect very small *p* values, reducing the likelihood of achieving statistical significance despite potential underlying effects.

In addition to reducing the statistical power of a study, missing data may introduce bias if not handled appropriately. However, in the present analysis, we used a quasi-experimental design and applied propensity score methods to balance the intervention and control groups on key baseline characteristics. By creating comparable groups, propensity score weighting helps mitigate confounding and reduces the risk that missing data will bias the results.

Additionally, we could not use the at-risk population of those with poor to moderate work ability (WAS score from 0 to 7) due to the limited sample size, or a 2-point increase in work ability as the primary outcome, as only 7.3% of participants achieved such an improvement during follow-up. Of those, approximately one-quarter had missing baseline data on covariates. Limiting the analysis to participants at risk at baseline further reduced the number of cases with a 2-point improvement, further compromising statistical power. Moreover, data on work ability and many favorable exposure changes were based on self-reports, which are subject to bias although validated measures were used. Work ability and job effort were also assessed using single items, limiting the depth of the measurements.

Finally, the generalizability of the study findings is limited and the benefits of the observed changes in risk factors may not apply to all midlife and older workers. The sample consisted mainly of female social and health care employees, which may limit the generalizability of the findings to other occupational groups. For example, evidence suggests that public and private sector employees differ, for example in terms of sickness absence rates (Hartikainen et al. 2022). Therefore, it is possible that results could be different if changes in risk factors were evaluated among private sector employees in other occupations. Also, we were not able to evaluate the changes in different subgroups (e.g. among genders, age groups or across the socioeconomic strata) due to the limited sample size.

## Conclusions

Positive changes in modifiable health- and work-related risk factors can improve the work ability of midlife and older social and health care employees. Changes in health-related factors associated with improved work ability were increasing physical activity, quitting smoking, decreasing alcohol usage, improving sleep quality and reducing psychological distress. Furthermore, reducing job demands and increasing job control and job rewards were work-related factors associated with improved work ability. These factors are essential to be considered in designing and implementing multidimensional interventions to enable prolonged working life participation of older workers. However, due to the observational study design, no causal inference can be determined.

## Data Availability

Data supporting this study cannot be made available due to ethical and legal restrictions.

## References

[CR1] Ahlstrom L, Grimby-Ekman A, Hagberg M, Dellve L (2010) The work ability index and single-item question: associations with sick leave, symptoms, and health–a prospective study of women on long-term sick leave. Scand J Work Environ Health 36(5):404–412. 10.5271/sjweh.2917 20372766 10.5271/sjweh.2917

[CR2] Ala-Mursula L, Vahtera J, Pentti J, Kivimaki M (2004) Effect of employee worktime control on health: a prospective cohort study. Occup Environ Med 61(3):254–261. 10.1136/oem.2002.005983 14985521 10.1136/oem.2002.005983 PMC1740736

[CR3] Alavinia SM, de Boer AG, van Duivenbooden JC, Frings-Dresen MH, Burdorf A (2009) Determinants of work ability and its predictive value for disability. Occup Med (Lond) 59(1):32–37. 10.1093/occmed/kqn148 19073989 10.1093/occmed/kqn148

[CR4] Andersen LN, Juul-Kristensen B, Roessler KK, Herborg LG, Sørensen TL, Søgaard K (2015) Efficacy of “Tailored Physical Activity” on reducing sickness absence among health care workers: a 3-months randomised controlled trial. Man Ther 20(5):666–671. 10.1016/j.math.2015.04.017 25983237 10.1016/j.math.2015.04.017

[CR5] Andersen LL, Izquierdo M, Sundstrup E (2017) Overweight and obesity are progressively associated with lower work ability in the general working population: cross-sectional study among 10,000 adults. Int Arch Occup Environ Health 90(8):779–787. 10.1007/s00420-017-1240-0 28660321 10.1007/s00420-017-1240-0

[CR6] Andersen LL, Pedersen J, Sundstrup E, Thorsen SV, Rugulies R (2021) High physical work demands have worse consequences for older workers: prospective study of long-term sickness absence among 69 117 employees. Occup Environ Med 78(11):829–834. 10.1136/oemed-2020-107281 33972376 10.1136/oemed-2020-107281 PMC8526881

[CR7] Bluher M (2020) Metabolically healthy obesity. Endocr Rev. 10.1210/endrev/bnaa004 10.1210/endrev/bnaa004 PMC7098708 32128581

[CR8] Camerino D, Conway PM, Sartori S, Campanini P, Estryn-Behar M, van der Heijden BI, Costa G (2008) Factors affecting work ability in day and shift-working nurses. Chronobiol Int 25(2):425–442. 10.1080/07420520802118236 18484372 10.1080/07420520802118236

[CR9] Canivet C, Choi B, Karasek R, Moghaddassi M, Staland-Nyman C, Ostergren PO (2013) Can high psychological job demands, low decision latitude, and high job strain predict disability pensions? A 12-year follow-up of middle-aged Swedish workers. Int Arch Occup Environ Health 86(3):307–319. 10.1007/s00420-012-0766-4 22476722 10.1007/s00420-012-0766-4

[CR10] Carlsson E, Hemmingsson T, Almroth M, Falkstedt D, Kjellberg K, Thern E (2024) Mediating effect of working conditions on the association between education and early labour market exit: a cohort study of Swedish men. Occup Environ Med 81(11):547–555. 10.1136/oemed-2024-109594 39586667 10.1136/oemed-2024-109594 PMC11671893

[CR11] De Bortoli MM, Oellingrath IM, Fell AKM, Burdorf A, Robroek SJW (2021) Influence of lifestyle risk factors on work ability and sick leave in a general working population in Norway: a 5-year longitudinal study. BMJ Open 11(2):e045678. 10.1136/bmjopen-2020-045678 10.1136/bmjopen-2020-045678 PMC7925900 33550269

[CR12] Elovainio M, Laaksonen M, Sakari K, Aalto AM, Jaaskelainen T, Rissanen H, Koskinen S (2022) Association of short poor work ability measure with increased mortality risk: a prospective multicohort study. BMJ Open 12(12):e065672. 10.1136/bmjopen-2022-065672 10.1136/bmjopen-2022-065672 PMC9791446 36549734

[CR13] Ervasti J, Pentti J, Seppala P, Ropponen A, Virtanen M, Elovainio M, Chandola T, Kivimaki M, Airaksinen J (2023) Prediction of bullying at work: a data-driven analysis of the Finnish public sector cohort study. Soc Sci Med 317:115590. 10.1016/j.socscimed.2022.115590 36463685 10.1016/j.socscimed.2022.115590

[CR14] Gan Y, Yang C, Tong X, Sun H, Cong Y, Yin X, Li L, Cao S, Dong X, Gong Y, Shi O, Deng J, Bi H, Lu Z (2015) Shift work and diabetes mellitus: a meta-analysis of observational studies. Occup Environ Med 72(1):72–78. 10.1136/oemed-2014-102150 25030030 10.1136/oemed-2014-102150

[CR15] Goldberg DP, Gater R, Sartorius N, Ustun TB, Piccinelli M, Gureje O, Rutter C (1997) The validity of two versions of the GHQ in the WHO study of mental illness in general health care. Psychol Med 27(1):191–197. 10.1017/s0033291796004242 9122299 10.1017/s0033291796004242

[CR16] Hale L, Singer L, Barnet JH, Peppard PE, Hagen EW (2017) Associations between midlife insomnia symptoms and earlier retirement. Sleep Health 3(3):170–177. 10.1016/j.sleh.2017.03.003 28526254 10.1016/j.sleh.2017.03.003 PMC7921848

[CR17] Harkonmaki K, Korkeila K, Vahtera J, Kivimaki M, Suominen S, Sillanmaki L, Koskenvuo M (2007) Childhood adversities as a predictor of disability retirement. J Epidemiol Commun Health 61(6):479–484. 10.1136/jech.2006.052670 10.1136/jech.2006.052670 PMC2465717 17496255

[CR18] Hartikainen E, Solovieva S, Viikari-Juntura E, Leinonen T (2022) Associations of employment sector and occupational exposures with full and part-time sickness absence: random and fixed effects analyses on panel data. Scand J Work Environ Health 48(2):148–157. 10.5271/sjweh.4003 34850957 10.5271/sjweh.4003 PMC9045233

[CR19] Ilmarinen J, Tuomi K (1992) Work ability of aging workers. Scand J Work Environ Health 18(Suppl 2):8–10 1514096

[CR20] Ilmarinen J, Tuomi K (2004) Past, present and future of work ability. People Work Res Rep 65:1–25

[CR21] Jaaskelainen A, Kausto J, Seitsamo J, Ojajarvi A, Nygard CH, Arjas E, Leino-Arjas P (2016) Work ability index and perceived work ability as predictors of disability pension: a prospective study among Finnish municipal employees. Scand J Work Environ Health 42(6):490–499. 10.5271/sjweh.3598 27706492 10.5271/sjweh.3598

[CR22] Juhola J, Arokoski JPA, Ervasti J, Kivimaki M, Vahtera J, Myllyntausta S, Saltychev M (2021) Internal consistency and factor structure of Jenkins Sleep Scale: cross-sectional cohort study among 80 000 adults. BMJ Open 11(1):e043276. 10.1136/bmjopen-2020-043276 10.1136/bmjopen-2020-043276 PMC7813292 33462100

[CR23] Juvani A, Oksanen T, Salo P, Virtanen M, Kivimaki M, Pentti J, Vahtera J (2014) Effort-reward imbalance as a risk factor for disability pension: the Finnish Public Sector Study. Scand J Work Environ Health 40(3):266–277. 10.5271/sjweh.3402 24247977 10.5271/sjweh.3402

[CR24] Kinnunen U, Nätti J (2018) Work ability score and future work ability as predictors of register-based disability pension and long-term sickness absence: a three-year follow-up study. Scand J Public Health 46(3):321–330. 10.1177/1403494817745190 29212430 10.1177/1403494817745190

[CR25] Kouvonen A, Kivimaki M, Vaananen A, Heponiemi T, Elovainio M, Ala-Mursula L, Virtanen M, Pentti J, Linna A, Vahtera J (2007) Job strain and adverse health behaviors: the Finnish Public Sector Study. J Occup Environ Med 49(1):68–74. 10.1097/JOM.0b013e31802db54a 17215715 10.1097/JOM.0b013e31802db54a

[CR26] Lahelma E, Laaksonen M, Lallukka T, Martikainen P, Pietilainen O, Saastamoinen P, Gould R, Rahkonen O (2012) Working conditions as risk factors for disability retirement: a longitudinal register linkage study. BMC Public Health 12:309. 10.1186/1471-2458-12-309 22537302 10.1186/1471-2458-12-309 PMC3438015

[CR27] Lallukka T, Shiri R (2020) Use of pseudo-trials in public health research: a case for propensity score matching. Eur J Public Health 30(3):393–394. 10.1093/eurpub/ckz241 32531038 10.1093/eurpub/ckz241

[CR28] Lallukka T, Overland S, Haaramo P, Saastamoinen P, Bjorvatn B, Sivertsen B (2014) The joint contribution of pain and insomnia to sickness absence and disability retirement: a register-linkage study among Norwegian and Finnish employees. Eur J Pain 18(6):883–892. 10.1002/j.1532-2149.2013.00432.x 24338923 10.1002/j.1532-2149.2013.00432.x

[CR29] Lau B (2008) Effort-reward imbalance and overcommitment in employees in a Norwegian municipality: a cross sectional study. J Occup Med Toxicol 3:9. 10.1186/1745-6673-3-9 18447923 10.1186/1745-6673-3-9 PMC2405796

[CR30] Leijon O, Balliu N, Lundin A, Vaez M, Kjellberg K, Hemmingsson T (2017) Effects of psychosocial work factors and psychological distress on self-assessed work ability: a 7-year follow-up in a general working population. Am J Ind Med 60(1):121–130. 10.1002/ajim.22670 27779327 10.1002/ajim.22670

[CR31] Leijten FR, de Wind A, van den Heuvel SG, Ybema JF, van der Beek AJ, Robroek SJ, Burdorf A (2015) The influence of chronic health problems and work-related factors on loss of paid employment among older workers. J Epidemiol Community Health 69(11):1058–1065. 10.1136/jech-2015-205719 26112957 10.1136/jech-2015-205719

[CR32] Lundin A, Leijon O, Vaez M, Hallgren M, Torgen M (2017) Predictive validity of the work ability index and its individual items in the general population. Scand J Public Health 45(4):350–356. 10.1177/1403494817702759 28385066 10.1177/1403494817702759

[CR33] Morelock JC, McNamara TK, James JB (2017) Workability and requests for flexible work arrangements among older adults: the role of a time and place management intervention. J Appl Gerontol 36(11):1370–1392. 10.1177/0733464815624149 26769823 10.1177/0733464815624149

[CR34] Morois S, Lemogne C, Leclerc A, Limosin F, Goldberg S, Goldberg M, Herquelot E, Zins M (2016) More than light alcohol consumption predicts early cessation from employment in French middle-aged men. Alcohol Alcohol 51(2):224–231. 10.1093/alcalc/agv092 26271114 10.1093/alcalc/agv092

[CR35] Natti J, Oinas T, Anttila T (2015) Time pressure, working time control and long-term sickness absence. Occup Environ Med 72(4):265–270. 10.1136/oemed-2014-102435 25564544 10.1136/oemed-2014-102435

[CR36] Nijp HH, Beckers DG, Geurts SA, Tucker P, Kompier MA (2012) Systematic review on the association between employee worktime control and work-non-work balance, health and well-being, and job-related outcomes. Scand J Work Environ Health 38(4):299–313. 10.5271/sjweh.3307 22678492 10.5271/sjweh.3307

[CR37] OECD. (2024b). Promoting Better Career Choices for Longer Working Lives: Stepping Up Not Stepping Out, Ageing and Employment Policies, OECD Publishing, Paris, 10.1787/1ef9a0d0-en

[CR38] Palmlof L, Skillgate E, Talback M, Josephson M, Vingard E, Holm LW (2019) Poor work ability increases sickness absence over 10 years. Occup Med (Lond) 69(5):359–365. 10.1093/occmed/kqz083 31219583 10.1093/occmed/kqz083

[CR39] Piantella S, Dragano N, McDonald SJ, Wright BJ (2021) Depression symptoms mediate the association between workplace stress and interleukin 6 in women, but not men: the Whitehall II study. Brain Behav Immun Health 12:100215. 10.1016/j.bbih.2021.100215 34589736 10.1016/j.bbih.2021.100215 PMC8474445

[CR40] de Rijk A (2013) Work disability theories: a taxonomy for researchers. Handbook of work disability, pp. 475–499

[CR41] Robroek SJ, Schuring M, Croezen S, Stattin M, Burdorf A (2013) Poor health, unhealthy behaviors, and unfavorable work characteristics influence pathways of exit from paid employment among older workers in Europe: a four year follow-up study. Scand J Work Environ Health 39(2):125–133. 10.5271/sjweh.3319 22949091 10.5271/sjweh.3319

[CR42] Ross ME, Kreider AR, Huang YS, Matone M, Rubin DM, Localio AR (2015) Propensity score methods for analyzing observational data like randomized experiments: challenges and solutions for rare outcomes and exposures. Am J Epidemiol 181(12):989–995. 10.1093/aje/kwu469 25995287 10.1093/aje/kwu469

[CR43] Salonen P, Arola H, Nygard CH, Huhtala H, Koivisto AM (2003) Factors associated with premature departure from working life among ageing food industry employees. Occup Med (Lond) 53(1):65–68. 10.1093/occmed/kqg012 12576568 10.1093/occmed/kqg012

[CR44] Salonsalmi A, Laaksonen M, Lahelma E, Rahkonen O (2012) Drinking habits and disability retirement. Addiction 107(12):2128–2136. 10.1111/j.1360-0443.2012.03976.x 22697358 10.1111/j.1360-0443.2012.03976.x

[CR45] Shiri R, Hiilamo A, Pietilainen O, Manty M, Rahkonen O, Lallukka T (2020a) Favourable changes in physical working conditions and the risk of all-cause sickness absence: a pseudo-experiment. Eur J Public Health 30(2):253–259. 10.1093/eurpub/ckz175 31578547 10.1093/eurpub/ckz175

[CR46] Shiri R, Lallukka T, Rahkonen O, Leino-Arjas P (2020b) Excess body mass and leisure time physical activity in the incidence and persistence of chronic pain. Pain Med 21(11):3094–3101. 10.1093/pm/pnaa102 32374375 10.1093/pm/pnaa102

[CR47] Shiri R, Hakola T, Harma M, Ropponen A (2021a) The associations of working hour characteristics with short sickness absence among part- and full-time retail workers. Scand J Work Environ Health 47(4):268–276. 10.5271/sjweh.3952 33755187 10.5271/sjweh.3952 PMC8091071

[CR48] Shiri R, Karhula K, Turunen J, Koskinen A, Ropponen A, Ervasti J, Kivimäki M, Härmä M (2021b) The effect of using participatory working time scheduling software on employee well-being and workability: a cohort study analysed as a pseudo-experiment. Healthcare. 10.3390/healthcare9101385 10.3390/healthcare9101385 PMC8544422 34683065

[CR49] Shiri R, Mattila-Holappa P, Kauppi M, Aalto V, Oksanen T, Ervasti J (2024) How does lowering psychosocial risks influence sickness absence? A prospective cohort study analyzed as a quasi-experiment. Eur J Public Health 34(1):136–142. 10.1093/eurpub/ckad211 38041444 10.1093/eurpub/ckad211 PMC10843950

[CR50] Silverstein M (2008) Meeting the challenges of an aging workforce. Am J Ind Med 51(4):269–280. 10.1002/ajim.20569 18271000 10.1002/ajim.20569

[CR51] Stenner HT, Eigendorf J, Kerling A, Kueck M, Hanke AA, Boyen J, Nelius A-K, Melk A, Boethig D, Bara C, Hilfiker A, Berliner D, Bauersachs J, Hilfiker-Kleiner D, Eberhard J, Stiesch M, Schippert C, Haverich A, Tegtbur U, Haufe S (2020) Effects of six month personalized endurance training on work ability in middle-aged sedentary women: a secondary analysis of a randomized controlled trial. J Occup Med Toxicol 15(1):8. 10.1186/s12995-020-00261-4 32391068 10.1186/s12995-020-00261-4 PMC7201966

[CR52] Tengland PA (2011) The concept of work ability. J Occup Rehabil 21(2):275–285. 10.1007/s10926-010-9269-x 21052807 10.1007/s10926-010-9269-x

[CR53] van de Ven D, Robroek SJ, Oude Hengel KM, van Zon SK, Brouwer S, Ots P, Burdorf A, Schuring M (2022) Associations of within-individual changes in working conditions, health behaviour and BMI with work ability and self-rated health: a fixed effects analysis among Dutch workers. BMJ Open 12(4):e058574. 10.1136/bmjopen-2021-058574 10.1136/bmjopen-2021-058574 PMC9058761 35487715

[CR54] van den Berg TI, Elders LA, de Zwart BC, Burdorf A (2009) The effects of work-related and individual factors on the work ability index: a systematic review. Occup Environ Med 66(4):211–220. 10.1136/oem.2008.039883 19017690 10.1136/oem.2008.039883

[CR55] van Dinter R, Jenks AC, Roels EH, Post MWM, Reneman MF (2025) Test-retest reliability and agreement of the work ability index-single item in persons with physical disabilities. Arch Phys Med Rehabil 106(7):1126–1130. 10.1016/j.apmr.2024.10.018 39571742 10.1016/j.apmr.2024.10.018

[CR56] Vuori J, Tornroos K, Ruokolainen M, Wallin M (2019) Enhancing late-career management among aging employees - a randomized controlled trial. J Vocat Behav 115:103327. 10.1016/j.jvb.2019.103327

[CR57] Wang XS, Armstrong ME, Cairns BJ, Key TJ, Travis RC (2011) Shift work and chronic disease: the epidemiological evidence. Occup Med (Lond) 61(2):78–89. 10.1093/occmed/kqr001 21355031 10.1093/occmed/kqr001 PMC3045028

[CR58] Xu S, Ross C, Raebel MA, Shetterly S, Blanchette C, Smith D (2010) Use of stabilized inverse propensity scores as weights to directly estimate relative risk and its confidence intervals. Value Health 13(2):273–277. 10.1111/j.1524-4733.2009.00671.x 19912596 10.1111/j.1524-4733.2009.00671.x PMC4351790

